# Relative abdominal adiposity is associated with chronic low back pain: a preliminary explorative study

**DOI:** 10.1186/s12889-016-3357-6

**Published:** 2016-08-02

**Authors:** Cristy Brooks, Jason C. Siegler, Paul W. M. Marshall

**Affiliations:** 1 School of Science and Health, Western Sydney University, Locked Bag 1797, Campbelltown Campus, Sydney, NSW 2751 Australia; 2 School of Science and Health, Western Sydney University, Campbelltown Campus, Sydney, Australia

**Keywords:** Chronic low back pain, Obesity, Abdominal adiposity, Ultrasound, Pain, Disability

## Abstract

**Background:**

Although previous research suggests a relationship between chronic low back pain (cLBP) and adiposity, this relationship is poorly understood. No research has explored the relationship between abdominal-specific subcutaneous and visceral adiposity with pain and disability in cLBP individuals. The aim of this study therefore was to examine the relationship of regional and total body adiposity to pain and disability in cLBP individuals.

**Methods:**

A preliminary explorative study design of seventy (*n* = 70) adult men and women with cLBP was employed. Anthropometric and adiposity measures were collected, including body mass index, waist-to-hip ratio, total body adiposity and specific ultrasound-based abdominal adiposity measurements. Self-reported pain and disability were measured using a Visual Analogue Scale (VAS) and the Oswestry Disability Index (ODI) questionnaires respectively. Relationships between anthropometric and adiposity measures with pain and disability were assessed using correlation and regression analyses.

**Results:**

Significant correlations between abdominal to lumbar adiposity ratio (A-L) variables and the waist-to-hip ratio with self-reported pain were observed. A-L variables were found to predict pain, with 9.1–30.5 % of the variance in pain across the three analysis models explained by these variables. No relationships between anthropometric or adiposity variables to self-reported disability were identified.

**Conclusions:**

The findings of this study indicated that regional distribution of adiposity via the A-L is associated with cLBP, providing a rationale for future research on adiposity and cLBP.

## Background

cLBP places a large economic burden on society, with loss of income and treatment costs in Australia in excess of $9 billion annually [1]. Low back pain (LBP) affects 10 % of the global population and is ranked as the 7th leading disability in the world and the highest ranked for years lived with the disability [2]. Obesity is also a costly and prevalent health condition, which has been previously linked to cLBP [3–13]. In the past this relationship has been demonstrated using body mass index (BMI) as a measure of obesity [3, 6, 8, 10, 14], which has been defined as an individual’s body weight divided by their height squared [15]. Despite its common use, the simplicity of BMI and its disregard for body composition [12] have led to its criticism and greater emphasis on alternative obesity measurements. This shift in focus is important because research suggests that adipose tissue may be of consequence in the pathogenesis of chronic pain conditions [12]. For example, increased adiposity (total body, upper and lower limbs, trunk, android and gynoid) is associated with higher levels of LBP intensity and disability [12]. Ultrasound (US) may be a suitable substitute for BMI and other simplistic obesity measurements as it is a valid and reliable measurement tool of assessing adiposity when compared to gold standard methods [16–21]. However, US has not yet been utilized in cLBP research.

Although there is an established relationship between adiposity and low back pain [12], the inconsistent and poorly defined terminology used in the past makes previous research confusing and difficult to draw conclusions from. Moreover, there is a lack of research on the distribution of adiposity and its possible relationship with pain and disability levels in cLBP. No studies have investigated whether regionally accumulated abdominal adiposity may be of more relevance than total body adiposity in a cLBP population. For example, visceral adiposity has been suggested to be more important than total adiposity in the risk of developing obesity-related disorders [20, 22]. Visceral adiposity has also been suggested to be of greater consequence to the metabolic profile [16, 23] and various medical pathologies [24] than subcutaneous adipose tissue, on the basis of physiological and metabolic differences such as adipocyte size and lipolytic activity [25]. It may then be suggested that the distribution of excess visceral adipose tissue could also be associated with increased pain in cLBP individuals. Several plausible mechanisms for a cLBP-visceral adiposity relationship exist, including inflammatory processes occurring from adipose tissue or increased mechanical load on the lumbar spine and surrounding structures produced by excess adiposity [12]. However, the cLBP-obesity relationship remains largely unknown, since research on the relationship between adiposity, primarily visceral, and cLBP is lacking.

In the exploration of the relative importance of regional versus total body adiposity, it is reasonable to believe that greater accumulation of adipose tissue in the abdominal region when compared to the lumbar region may also be of significance in the relationship to pain and disability in cLBP. This abdominal to lumbar adiposity ratio may be important, as greater abdominal adiposity could have flow-on effects for cLBP sufferers beyond that of an increase in body weight. For example, increased abdominal adipose tissue may result in the adoption of a compensatory hyperlordotic posture to counteract the constant anterior flexion torque placed on the lumbar spine. This excess anterior mass is worthy of investigation, since an increase in compressive force may predispose the spine to injury [26]. Irrespective of the potential metabolic or biomechanical mechanisms that may be responsible for such a relationship, the parameters of a possible association between adiposity and cLBP should first be examined.

As a result of the inconsistencies of previous research and the potentially important consequences of visceral adiposity on the persistence of cLBP via metabolic factors such as the stimulation of inflammatory processes, it is warranted to examine the significance of adiposity distribution and particularly visceral adiposity on the obesity-cLBP relationship. US may then be employed to investigate the possible importance of visceral adipose tissue, since it has been shown to be a valid and reliable method of assessing abdominal adiposity [16–21]. Therefore, the aim of this study was to examine the relationship between regional and total body adiposity with pain and disability in cLBP individuals. The experimental objectives of this study were: 1) To use US-derived ratios to assess abdominal adipose tissue distribution in individuals with cLBP, 2) To perform correlation and regression analyses to examine relationships between anthropometric and adiposity variables with self-reported pain and disability in cLBP individuals, and 3) To perform the correlation and regression analyses on pain and disability subgroups within the cLBP dataset. The hypothesis of this study was that greater abdominal adiposity, particularly visceral, would be associated with increased self-reported pain and disability in a cLBP population.

## Methods

### Study design

A preliminary explorative study design was employed to examine the relationship between adiposity distribution with pain and disability in a cLBP population. All participant data was collected at a tertiary education facility in Western Sydney, Australia, over a three-year period with two cycles of participant recruitment and data collection.

### Study population

Seventy (*n* = 70) adult men and women aged 18–76 years were included in the study and were recruited through the use of media advertising and leaflet drops in the local area. All included participants had cLBP (pain between the costal margin and gluteal fold for a minimum of three months). Participants were excluded if they had a history of spinal surgery, spinal fracture, diagnosed lumbar disc herniation (and attained a positive result on the straight leg raise test), existing bone, cardiac or nervous system condition, diagnosed severe mental illness, severe postural abnormality, pain radiating below the knee or diagnosed inflammatory joint disease. Written informed consent was provided by all participants. This study had ethical approval for research on human subjects by the Human Research Ethics Committee review board on the basis of the Declaration of Helsinki.

### Anthropometric measures

Height, weight, waist circumference (WC), hip circumference, BMI and waist-to-hip ratio (WHR) were measured while participants were barefoot and wearing lightweight clothing. Height was measured using a wall-mounted stadiometer (Veeder-Root high speed counter, Elizabethtown, N.C.) and recorded to the nearest 0.1 cm. Weight was measured using a calibrated digital scale (A&D UC-321, A&D Co., Ltd) and recorded to the nearest 0.1 kg. Waist circumference was measured using an anthropometric tape measure (Lufkin Executive Diameter Pocket Tape W606PM) at the narrowest point between the costal margin and the iliac crest and recorded to the nearest 0.1 cm. Hip circumference was measured at the widest point of buttocks approximately level with the greater trochanters of the femur and recorded to the nearest 0.1 cm. BMI was calculated as weight divided by height squared (kg/m^2^) [15]. WHR was calculated as WC divided by hip circumference.

### Adiposity measures

#### Total body adiposity

Total body adiposity was measured using bioelectrical impedance analysis (BIA) (Metagenics VLA50, variation of ImpDF50, ImpediMed Limited, Eight Mile Plains, QLD, 2005), which has been shown to be a valid and reliable method when compared to gold standard methods [27–34]. Participants were required to refrain from food, drink and exercise 2 hours prior to the test and avoid alcohol in the 12 hours prior. Immediately prior to the test, participants emptied their bladder and lay supine on a plinth for 5 minutes to stabilise body fluids. The participant remained in this position with arms by their sides for the duration of the test. Pairs of electrodes (Ag/AgCl 3 cm diameter, Kendall Medi-Trace 100, Tyco Healthcare Group LP, Mansfield, MA) were placed on their hand and foot on the right side of the body. Prior to electrode placement, the skin was adequately prepared using a safety razor, fine abrasion tape and alcohol swabs to remove excess hair and reduce impedance. The hand electrodes were placed between the radial and ulna styloid processes 1 cm proximal to the metacarpophalangeal joint of the middle finger. The foot electrodes were placed between the medial and lateral malleoli of the tibia and fibula, respectively, and 1 cm proximal to the metatarsophalangeal joint of the middle toe. Each electrode pair was a minimum of 10 cm apart. Resistance and reactance was recorded from the BIA device and then used to calculate total body adiposity percentage from the BIA software.

#### Regional adiposity

**Fig. 1:**
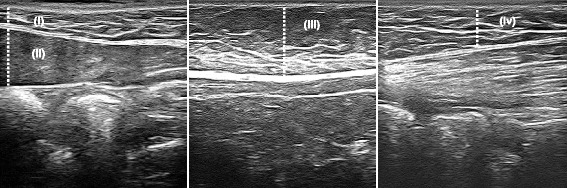
Examples of abdominal US measurements (i) minimum subcutaneous abdominal adiposity (ii) maximum pre-peritoneal abdominal adiposity (iii) maximum subcutaneous abdominal adiposity A (iv) maximum subcutaneous abdominal adiposity B

**Fig. 2:**
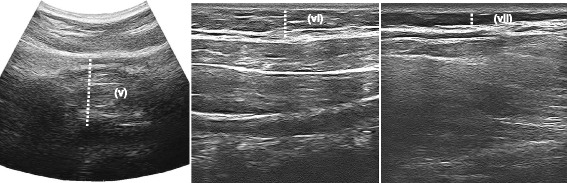
Examples of intra-abdominal, supra-iliac and lumbar US measurements (v) maximum intra-abdominal adiposity (vi) maximum subcutaneous supra-iliac adiposity (vii) maximum subcutaneous lumbar adiposity

Regional adiposity (including lumbar, supra-iliac and multiple abdominal sites) was measured with US using previously validated and reliable methods [16]. Five (5) subcutaneous adiposity and two (2) visceral adiposity measurements were conducted over five (5) anatomical locations on the surface of the skin in the trunk region of each participant, of which five (5) have been described elsewhere [16]. Details and images of each measurement are listed in Table 1 and shown in Figs. 1 and 2 respectively. Participants were required to lie supine for a period of 10 minutes prior to US testing to allow body fluids to stabilise. Each measurement required the use of conductive gel to gain a clear image.

**Table 1:** Ultrasound measurements

Measurement	Probe	Anatomical location	Method used for measurement
msA	Linear	Just below the xiphoid process of the sternum	Minimum distance between the fat-skin barrier and the anterior surface of the linea alba
MppA	Linear	Just below the surface of the xiphoid process of the sternum (same anatomical position as the minimum subcutaneous adiposity measurement)	Maximum distance between the posterior surface of the linea alba and the anterior surface of the peritoneum covering the liver
MsA	Linear	(A) 2 cm above the umbilicus and (B) 2 cm below the umbilicus	Maximum distance between the fat-skin barrier and the anterior surface of the linea alba
MiA	Convex	2 cm above the umbilicus (same anatomical position as maximum subcutaneous abdominal adiposity A)	Maximum distance between the posterior surface of the rectus abdominis muscle and the anterior wall of the abdominal aorta
MsSI	Linear	Just above the iliac crest on the mid-axillary line	Maximum distance between the fat-skin barrier and the anterior surface of the external oblique muscle
MsL	Linear	Level of L4/L5 directly over the lumbar erector spinae muscle	Maximum distance between the fat-skin barrier and the anterior surface of the lumbar erector spinae muscle

*MsA* minimum subcutaneous abdominal adiposity, *MppA* maximum pre-peritoneal abdominal adiposity, *MsA* maximum subcutaneous abdominal adiposity, *MiA* maximum intra-abdominal adiposity, *MsSI* maximum subcutaneous supra-iliac adiposity, *MsL* maximum subcutaneous lumbar adiposity

#### Adiposity ratios

The adiposity ratios calculated from ultrasound-derived adiposity thickness measurements are defined in Table 2, of which one has been previously described [16]. Such ratios were worthy of inclusion as past research has questioned simplistic anthropometric measurements such as BMI and WHR due to their lack of sensitivity and specificity [12, 35, 36]. Additionally, existing evidence implies a relationship between adiposity and pain that may be complex and multifactorial [12]. Consequently, the examination of adiposity relative to the individual may be crucial to better understanding the relationship between adiposity and cLBP.

**Table 2:** Ultrasound-derived adiposity variables

Measure	Calculation	Definition
A-L	(MsAa + MiA)/MsL	Abdominal-to-lumbar adiposity ratio (total subcutaneous and visceral abdominal adiposity thickness relative to lumbar adiposity thickness)
S-M	(MsAa + MsSI + MsL)/weight	Subcutaneous adiposity to mass ratio (total subcutaneous trunk adiposity thickness relative to overall body mass)
V-M	(MppA + MiA)/weight	Visceral adiposity to mass ratio (total visceral trunk adiposity thickness relative to overall body mass)
MAR-A	MsAa/MiA	Maximal abdominal ratio A (ratio between subcutaneous and visceral abdominal adiposity)
A-L/BMI	[(MsAa + MiA)/MsL]/[weight/(height x height)]	Abdominal-to-lumbar adiposity ratio to BMI (ratio between abdominal and lumbar adiposity thickness relative to overall body mass index
A-L/WHR	[(MsAa + MiA)/MsL]/(waist circumference/hip circumference)	Abdominal-to-lumbar adiposity ratio to WHR (ratio between abdominal and lumbar adiposity thickness relative to the ratio between waist and hip circumferences)
TC-TBA	(MsAa + MsSI + MsL)/total body adiposity percentage	Total circumference to total body adiposity ratio (total trunk circumference thickness relative to total body adiposity percentage)

*MppA* maximum pre-peritoneal abdominal adiposity, *MsAa* maximum subcutaneous abdominal adiposity A, *MiA* maximum intra-abdominal adiposity, *MsSI* maximum subcutaneous supra-iliac adiposity, *MsL* maximum subcutaneous lumbar adiposity

### Pain and disability

Self-reported pain was measured using a VAS, with ‘no pain’ on the left anchor and ‘worst pain imaginable’ on the right anchor, whereby the participant rated their current cLBP on a 100 mm line [37, 38]. Self-reported disability was measured using the ODI questionnaire, whereby participants filled in a 10-item questionnaire that was scored and converted to a percentage [37–39]. VAS and ODI have been previously shown to be valid and reliable methods of measuring self-reported pain and disability respectively in pain research, including cLBP populations [40–45].

### Statistical analysis

Statistical analyses were performed using SPSSv23 (IBM Corp., 2015). Mean and standard deviation were presented for characteristics of the study sample. Normal distribution of data was assessed by Kolmogorov-Smirnoff and Shapiro-Wilk tests, and examination of Q-Q plots, frequency histograms and standard errors of skewness and kurtosis. Variables not normally distributed were log transformed and parametric methods of analysis were then used. Three (3) datasets were used for statistical analysis; the total sample of participants (*n* = 70) to avoid the potential for detection bias, a VAS subgroup with a minimum level of pain as indicated by 2.0 or greater on the VAS scale (*n* = 42), and an ODI subgroup with a minimum level of disability as indicated by 10.0 % or greater on the ODI questionnaire (*n* = 52). Pearson correlation coefficients were used to identify relationships between anthropometric and adiposity variables with self-reported pain and disability. Stepwise regression analyses were performed to explain relationships between anthropometric and adiposity variables with pain and disability, as well as determine the proportion of variance in pain and disability explained by such variables. Adjusted R square values were reported for significant relationships. Predictor variables included in the regression analysis were determined by the results of the correlation analysis, where only variables found to be correlated with pain or disability were included in the regression models to reduce the potential effect of confounding variables. The variance inflation factor (VIF) was used to determine the effect of collinearity of prediction variables on regression analyses. A VIF > 5 for any two variables was used to indicate collinearity, in which case the variable with the higher VIF was removed from the prediction model. Missing data were addressed through exclusion of the incomplete variable/s for a given participant from the analysis model. The study size was arrived at with the use of post-hoc calculations of statistical power. Statistical significance was set at *p* < 0.05.

## Results

A total of *n* = 122 individuals were screened for inclusion and *n* = 70 cLBP individuals were eligible and chose to participate in the study. The characteristics of the study sample are summarised in Tables 3, 4 and 5. One (1) participant had missing data of the minimum subcutaneous lumbar adiposity measurement.

**Table 3:** Demographic characteristics of the study sample (*n* = 70)

Age (yrs)	39.57 ± 11.01
cLBP (yrs)	9.84 ± 8.60
Gender (M/F)	30 M, 40 F
Height (m)	1.70 ± 0.08
Weight (kg)	79.66 ± 17.44
BMI (kg/m^2^)	27.49 ± 5.63
WC (cm)	87.72 ± 14.68
HC (cm)	104.94 ± 10.03
WHR	0.83 ± 0.09
TBA	29.99 ± 10.87
ODI	16.66 ± 9.65
VAS	2.38 ± 1.78

SD data mean ±

*cLBP* chronic low back pain, *BMI* body mass index, *WC* waist circumference, *HC* hip circumference, *WHR* waist-to-hip ratio, *TBA* total body adiposity percentage, *ODI* oswestry disability index, *VAS* visual analogue scale

**Table 4:** Absolute ultrasound measurements (mm) of the study sample (*n* = 70)

msA	12.34 ± 7.79
MppA	13.36 ± 4.53
MsAa	20.19 ± 9.69
MsAb	19.60 ± 9.90
MiA	49.77 ± 23.01
MsSI	14.40 ± 7.69
MsL	8.36 ± 6.90

Data mean ± SD

*msA* minimum subcutaneous abdominal adiposity, *MppA* maximum pre-peritoneal abdominal adiposity, *MsAa* maximum subcutaneous abdominal adiposity A, *MsAb* maximum subcutaneous abdominal adiposity B, *MiA* maximum intra-abdominal adiposity, *MsSI* maximum subcutaneous supra-iliac adiposity, *MsL* maximum subcutaneous lumbar adiposity

**Table 5:** Relative ultrasound measurements and ratios of the study sample (*n* = 70)

A-L	12.42 ± 9.12
S-M	0.54 ± 0.24
V-M	0.78 ± 0.20
MAR-A	0.46 ± 0.25
A-L/BMI	0.47 ± 0.38
A-L/WHR	14.44 ± 10.11
TC-TBA	1.44 ± 0.52

Data mean ± SD

*A-L* abdominal to lumbar adiposity ratio, *S-M* subcutaneous adiposity to mass ratio, *V-M* visceral adiposity to mass ratio, *MAR-A* maximum abdominal ratio A, *A-L/BMI* abdominal to lumbar adiposity ratio to BMI, *A-L/WHR* abdominal to lumbar adiposity ratio to WHR, *TC-TBA* total circumference to total body adiposity ratio

### Relationship between anthropometric and adiposity measures to pain and disability

Correlations between anthropometric and adiposity measures with pain are shown in Table 6. There were no significant correlations observed between self-reported disability and anthropometric or adiposity variables in any of the analysis models. ODI was found to be correlated to VAS in the total sample (*r* = 0.264, *p* = 0.028), but not in either of the subgroup analysis models.

**Table 6:** Significant correlations between anthropometric and adiposity variables with self-reported pain

Analysis model	Variable	*r*	*p*
Total sample (*n* = 70)	A-L	0.323	0.007
	A-L/WHR	0.315	0.008
	A-L/BMI	0.303	0.011
VAS subgroup (*n* = 42)	A-L	0.566	<0.001
	A-L/WHR	0.568	<0.001
	A-L/BMI	0.546	<0.001
ODI subgroup (*n* = 52)	A-L	0.493	<0.001
	A-L/WHR	0.438	0.001
	A-L/BMI	0.5111	<0.001
	WHR	0.287	0.039

*VAS* visual analogue scale, *ODI* oswestry disability index, *A-L* abdominal to lumbar adiposity ratio, *A-L/WHR* abdominal to lumbar adiposity ratio to WHR, *A-L/BMI* abdominal to lumbar adiposity ratio to *BMI* WHR, waist-to-hip ratio

Stepwise regression showed that 9.1 % (*p* = 0.007) of the variance in pain was explained by A-L alone in the total sample analysis (*n* = 70), which was increased to 15.7 % (*p* = 0.001) when ODI was added to the model. Results of the stepwise regression for the VAS subgroup indicated that 30.5 % of the variance in pain could be explained by A-L/WHR (*p* < 0.001). Similar results were observed in the ODI subgroup regression analysis, as 24.7 % of the variance in pain was explained by A-L/BMI (*p* < 0.001). No regression analysis was performed on self-reported disability on the basis of no significant correlations to anthropometric or adiposity variables in any of the analysis models. Post-hoc results revealed an achieved statistical power of β = 0.75 for the variance in pain explained by A-L/WHR.

## Discussion

It was hypothesised that greater abdominal adiposity, particularly visceral, would be associated with increased self-reported pain and disability in cLBP individuals. This study’s findings showed a relationship between anthropometric and adiposity measures to self-reported pain in cLBP, but not disability. More specifically, A-L relative to the size of the individual was the best predictor of self-reported pain.

The results of this study support previous suggestions that visceral adiposity may be more important than subcutaneous adiposity in the relationship to pain. For example, the overflow of adipocytes into excess visceral and ectopic stores may initiate a process of metabolic dysfunction [46] resulting from the disrupted equilibrium between energy intake and lipid oxidation [36]. Consequently, this overflow may promote the release of adipocyte-derived pro-inflammatory cytokines [47] contributing to insulin resistance and end-stage disease [46, 47], but also to hyperalgesia and central sensitisation [48]. In addition to metabolic dysfunction, there is growing evidence for the pathophysiological consequences on bone and skeletal muscle integrity and function from abnormal lipid accumulation [36]. The result may then be chronic low-grade systemic inflammation [46, 47] and therefore the persistence of a chronic pain state [48]. For example, increased levels of C-reactive protein, a sensitive acute-phase protein associated with body adiposity measures [49], has been linked to greater odds of reporting LBP symptoms, particularly in those measured as obese by BMI or WC [49]. It has been suggested that increased C-reactive protein may be indicative of early signs of low-grade chronic systemic inflammation [49]. Consequently, it may validate the implication of pro-inflammatory cytokines in the complex pathways of musculoskeletal pain [49] and further support the use of visceral adiposity measurements, such as US, in the research of cLBP and other chronic pain pathologies.

This study’s findings may also support a theorised metabolic mediation in the adiposity-pain relationship [12]. Since pain was found to be significantly correlated with A-L relative to BMI or WHR, visceral adiposity relative to body size and shape may be an important consideration for future research. For example, the distribution of A-L may be just as important as the overall representation of body size and mass distribution. Therefore, it may be the accumulation of body mass coupled with greater levels of relative adiposity that puts an individual in an increased or more persistent cLBP state.

The moderate to strong correlations and prediction models between pain and A-L relative to WHR and BMI may advocate a possible physiological or biomechanical mediation between obesity and cLBP. For instance, WHR measures an individual’s anatomical circumference of the waist compared to the hips to assess adiposity distribution [50] and the associated risk of deviating from optimal body morphology for physical health. In turn, BMI is a measure of overall body size as a relative association between height and weight [15], with an optimum balance to achieve the ‘healthy’ range. Consequently, coupling WHR and BMI with the A-L/pain relationship may further support a physiological or biomechanical mediation. It is reasonable to believe that the body can only manage a degree of anterior-to-posterior load, but is also functionally limited by waist-to-hip load and overall body load. For example, perhaps an individual with a high A-L, large WHR and elevated BMI may be in greater pain than someone with the same A-L but lower WHR and BMI. It may be the accumulation of the overall body mass and weight distribution including adiposity that acts as a pain catalyst, but is the A-L that is most instrumental in observable and measurable biomechanical changes. For instance, it is plausible that greater anterior mass may result in increased compensatory lordosis during normal daily posture, manifest by spinal hyperextension, and thereby excess abdominal adiposity may result in increased magnitude or repetition of compression loading, which is a known precursor for risk of intervertebral disc injury [26]. Moreover, previous research suggests that both vertebral joint compression and postural deviation may impact upon shear injury potential [51]. Irrespective of these yet unconfirmed inferences, it is known that obesity and cLBP are linked [3–13] and that simplistic measurements like BMI are unrelated to cLBP [52] and lack the sensitivity to detect excessive adiposity in non-obese individuals [36]. Therefore, future research may need to explore more comprehensive measurements such as A-L to further quantify and explain the adiposity-cLBP relationship.

The hypothesis that greater abdominal adiposity would be associated with increased disability was not supported, as no correlations were found between anthropometric and adiposity variables with disability. This finding was not supported or refuted by previous research, since no other studies to the authors’ knowledge have examined the relationship between adiposity and disability associated with cLBP. An earlier study reporting a relationship between adiposity and disability associated with LBP [12] was not specific to cLBP and assessed adiposity and disability using different methods to those used in this study. Therefore, further research may be necessary to confirm that adiposity and disability are unrelated in cLBP.

The novelty of this research lends itself to potential constraints, such as the use of absolute and relative adiposity ratios not previously studied. The removal of variables to eliminate collinearity during statistical analysis may have excluded potentially relevant variables from the prediction models. However, any variables removed were those with the least impact on the prediction models. Correlation analysis between each variable with pain and disability also ensured all relevant relationships between variables were explored. It may be irrelevant which A-L variables were left in the regression analyses, since all A-L variables were found to have strong correlations to pain. The use of WHR instead of WC may be a limitation since adipose tissue deposits in the abdominal versus gluteofemoral region may have different biological mechanisms and therefore altered health risk implications [50]. For this reason, future studies into the A-L/cLBP relationship may benefit more from the use of WC instead of WHR. The selection of VAS and ODI cutoff values may have excluded potentially relevant data, but since the majority of existing research explored the minimum level of clinically meaningful change over time no previous consensus on normative scores for minimal pain or disability levels in cLBP was found. Therefore, values were set from collaborative evidence of minimal important change values in VAS ranging from 1.5–2.0 [53] and a normative score of 10.19 for ODI of ‘normal’ populations [42], which was deemed appropriate based on available evidence. The study results can only be generalised to adult cLBP populations.

## Conclusions

The results of this study demonstrated significant relationships between abdominal adiposity and cLBP. A-L combined with increased WHR and BMI was a predictor of pain variance. Therefore, an individual’s adiposity distribution relative to their body or trunk mass may be of greater importance in the cLBP-obesity relationship than single measurements alone. These findings support the use of US-based methodologies for future cLBP research. Until the mechanisms responsible for the adiposity-cLBP relationship are better understood, attempts to manipulate it through pain or adiposity reduction treatment may be of little benefit. For this reason, additional research into possible physiological, metabolic and biomechanical mediators between adiposity distribution and pain manifestation in cLBP is warranted.

## Abbreviations

A-L, abdominal to lumbar adiposity ratio; A-L/WHR, abdominal to lumbar adiposity ratio relative to waist-to-hip ratio; A-L/BMI, abdominal to lumbar adiposity ratio relative to body mass index; BIA, bioelectrical impedance analysis; BMI, body mass index; cLBP, chronic low back pain; LBP, Low back pain; ODI, Oswestry Disability Index; US, ultrasound; VAS, visual analogue scale; VIF, variance inflation factor; WC, waist circumference; WHR, waist-to-hip ratio
